# Quantitative phosphoproteomic analysis reveals involvement of PD-1 in multiple T cell functions

**DOI:** 10.1074/jbc.RA120.014745

**Published:** 2021-01-13

**Authors:** Anna S. Tocheva, Michael Peled, Marianne Strazza, Kieran R. Adam, Shalom Lerrer, Shruti Nayak, Inbar Azoulay-Alfaguter, Connor J.R. Foster, Elliot A. Philips, Benjamin G. Neel, Beatrix Ueberheide, Adam Mor

**Affiliations:** 1 Columbia Center for Translational Immunology, Columbia University Medical Center, New York, USA; 2 Department of Genetics and Genomic Sciences, Icahn School of Medicine at Mount Sinai, New York, New York, USA; 3 Perlmutter Cancer Center, NYU School of Medicine, New York, New York, USA; 4 The Institute of Pulmonary Medicine, the Chaim Sheba Medical Center, Ramat Gan, Israel; 5 Division of Rheumatology, Columbia University Medical Center, New York, New York, USA; 6 Proteomics Laboratory, NYU School of Medicine, New York, New York, USA; 7 Department of Biochemistry and Molecular Pharmacology, NYU School of Medicine, New York, New York, USA

**Keywords:** T cell, programmed cell death protein 1 (PD-1), programmed cell death ligand 2 (PD-L2), phosphoproteomics, cell signaling, mass spectrometry, signaling networks, immunology, T cell receptor (TCR), inhibition mechanism, immunotherapy, kinase-substrate relationships

## Abstract

Programmed cell death protein 1 (PD-1) is a critical inhibitory receptor that limits excessive T cell responses. Cancer cells have evolved to evade these immunoregulatory mechanisms by upregulating PD-1 ligands and preventing T cell–mediated anti-tumor responses. Consequently, therapeutic blockade of PD-1 enhances T cell–mediated anti-tumor immunity, but many patients do not respond and a significant proportion develop inflammatory toxicities. To improve anti-cancer therapy, it is critical to reveal the mechanisms by which PD-1 regulates T cell responses. We performed global quantitative phosphoproteomic interrogation of PD-1 signaling in T cells. By complementing our analysis with functional validation assays, we show that PD-1 targets tyrosine phosphosites that mediate proximal T cell receptor signaling, cytoskeletal organization, and immune synapse formation. PD-1 ligation also led to differential phosphorylation of serine and threonine sites within proteins regulating T cell activation, gene expression, and protein translation. *In silico* predictions revealed that kinase/substrate relationships engaged downstream of PD-1 ligation. These insights uncover the phosphoproteomic landscape of PD-1–triggered pathways and reveal novel PD-1 substrates that modulate diverse T cell functions and may serve as future therapeutic targets. These data are a useful resource in the design of future PD-1–targeting therapeutic approaches.

The immune system utilizes a myriad of inhibitory mechanisms to prevent excessive T cell responses, including surface-expressed inhibitory receptors, also termed immune checkpoints (1). Cancer cells evolve to evade these inhibitory receptor pathways to prevent T cell–mediated tumor clearance by expressing surface ligands that bind these receptors. Recent years have seen the development and implementation of immune checkpoint therapies, which block inhibitory receptors on T cells or their ligands on tumor cells, to augment anti-tumor immune responses (2, 3, 4). The PD-1 pathway is an important immune checkpoint for T cells (5). Antibodies that block PD-1 function promote T cell–mediated identification and clearance of tumor cells and are powerful therapeutics against a wide variety of cancers (6). Unfortunately, only a fraction of the patients who receive anti-PD-1 therapeutics have durable clinical responses, and more than 30% of responders develop immune-related adverse events (7). Better understanding of the signaling pathways triggered by PD-1 would aid in the development of new agents to target proteins downstream of PD-1 and augment the activity of PD-1–directed therapeutics.

Despite its clinical utility, the molecular pathways engaged by PD-1 remain poorly defined. PD-1 lacks intrinsic enzymatic activity and instead recruits other proteins to mediate its inhibitory function. In T cells, T cell receptor (TCR) recognition of antigens presented by major histocompatibility complex (MHC) molecules and PD-1 colocalization with the TCR are absolute requirements for PD-1 function (8). Following antigen recognition, PD-1 binds to its ligands, programmed death ligand 1 (PD-L1) (9) and programmed death ligand 2 (PD-L2) (10), expressed on tumor cells and antigen-presenting cells, respectively. Ligand binding leads to tyrosine phosphorylation of the immune tyrosine inhibitory motif and immune tyrosine switch motif within the cytoplasmic tail of PD-1, which subsequently recruits the tyrosine phosphatase Src homology 2 (SH2) domain containing tyrosine phosphatase 2 (SHP-2) (8). In turn, SHP-2 dephosphorylates tyrosines within proteins critical for TCR signaling, such as CD3ζ, ZAP70 (11), CD28 (12), and C3G (12). PD-1 ligation has also been demonstrated to interfere with cell cycle progression and T cell proliferation by indirectly inhibiting signaling mediated by MEK-ERK and AKT (13, 14, 15).

Our knowledge regarding the phosphorylation networks that are triggered by PD-1 and that restrict proximal and distal TCR signaling is limited. This significant knowledge gap undermines our understanding of PD-1 function in T cells and hampers the development of improved therapeutics targeting the PD-1 axis. To address this, we combined isobaric labeling with phosphopeptide enrichment and MS to obtain an unbiased global overview of the PD-1–regulated phosphoproteome. To meet the challenge of large cell numbers required for this analysis and the significant variability of PD-1 expression and signaling between different human T cell subsets, we carried out phosphoproteomic interrogation in Jurkat T cells. Our reductionist approach combined with the extensive TCR signaling studies carried in this cell line (16) allowed us to first reconstruct the signaling pathways engaged by PD-1 and, after that, to validate the functional consequences of PD-1 ligation following T cell activation using primary human T cells. Through this approach we identified that the breadth of PD-1–regulated phosphorylation networks extends far beyond the expected proximal TCR signaling. In fact, the functional consequences of PD-1 signaling in T cells are at the intersect of PD-1–regulated phosphorylation networks that are specifically associated with TCR signaling, cytoskeletal organization, cell cycle, gene expression, and protein translation. These data provide novel insights into PD-1–triggered signaling networks and the role of this critical inhibitory receptor in T cell activation and downstream responses. These data will serve as a springboard in the development of novel PD-1 pathway–targeting therapeutics.

## Results

### PD-1 signaling leads to global differential phosphorylation of tyrosine, serine, and threonine sites

We investigated the PD-1–regulated phosphoproteome in response to PD-L2 ligation. Our choice of PD-L2 *versus* PD-L1 was dictated by the higher affinity of PD-L2 for PD-1 and greater inhibition of Interleukin-2 (IL-2) production (Fig. S1A) and CD3ζ Y142 immunoreceptor tyrosine-based activation motif (ITAM) phosphorylation (Fig. S1B) in Jurkat T cells. We also did not ligate CD28 because our aim was to identify PD-1–triggered signaling pathways that may be engaged within the tumor, and tumor cells do not express B7.1 and B7.2, the ligands for CD28. Additionally, repulsive guidance molecule b (RGMb) and CD80, which have been also shown to bind PD-L2 and PD-L1, respectively, were not expressed by Jurkat T cells (Fig. S1C). Finally, in a previous study we were unable to demonstrate significant functional differences between PD-L1 and PD-L2 following PD-1 ligation in Jurkat T cells (17). Consequently, we reasoned that PD-L2 will elicit a response with a higher magnitude compared with PD-L1. To investigate PD-1–regulated T cell phosphoproteome, we treated Jurkat T cells with beads coated with anti-human CD3 antibody (αCD3), anti-human CD3 antibody together with a recombinant human PD-L2-Fc (αCD3+PD-L2), or an IgG1 isotype control antibody (unstimulated) for 30 s, 5 min, or 15 min. Although resting Jurkat T cells have low PD-1 surface expression (Fig. S1D), and as shown by other investigators, it was sufficient to elicit decreased phosphorylation of multiple tyrosine residues as early as 30 s after treatment with αCD3+PD-L2 (Fig. S1E). These preliminary analyses confirmed that the majority of early TCR proximal signaling events occurred within 5 min of TCR crosslinking, and therefore our downstream analyses are performed at 30 s and 5 min.

**Figure 1:**
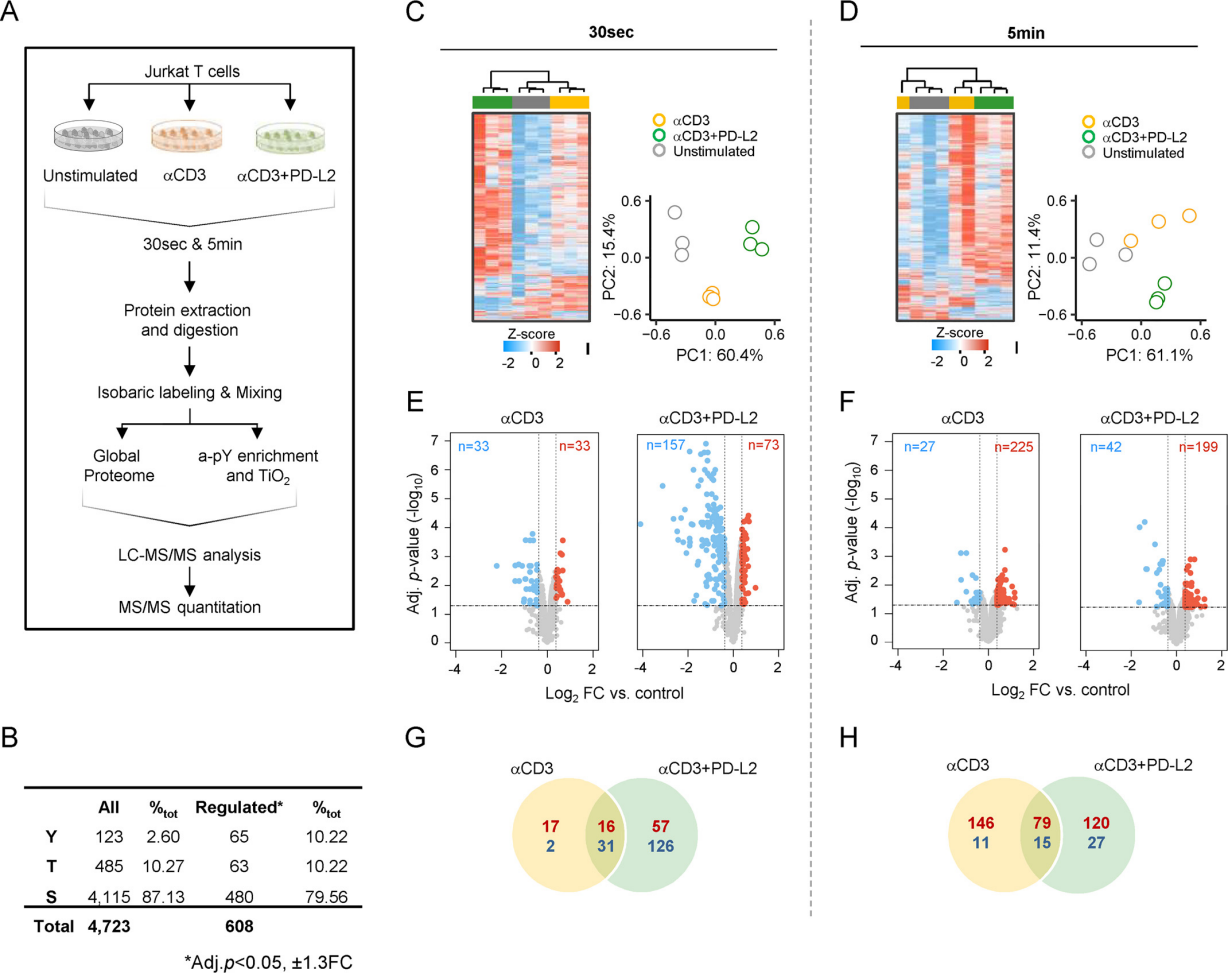
**Experimental design and global overview of the PD-1–regulated phosphoproteome.***A*, experimental workflow. *B*, quantified total and differentially phosphorylated phosphosites. *C* and *D*, cluster analysis and principal component plots of normalized intensities for all phosphopeptides at 30 s (*C*) and 5 min (*D*) post-stimulation. *E* and *F*, volcano plots of differentially increased (*red*) or decreased (*blue*) phosphosites relative to the unstimulated control in response to αCD3 or αCD3+PD-L2 stimulation at 30 s (*E*) and 5 min (*F*). *Horizontal dotted lines* correspond to –Log10 adjusted *p*-value ≤ 0.05, and *vertical lines* correspond to FC ≥ ±1.3. *G* and *H*, number of shared and treatment-specific increased (*red*) or decreased (*blue*) phosphosites at 30 s (*G*) and 5 min (*H*).

To determine the PD-1–regulated phosphoproteome following PD-L2 binding in Jurkat T cells, we used tandem mass tag spectrometry (TMT) incorporating multiplexed isobaric labeling and phosphopeptide enrichment for phosphorylated serine (pSer), threonine (pThr), and tyrosine (pTyr) sites (Fig. 1*A*). We identified a total of 4,723 phosphosites from 1,686 proteins (Fig. 1*B*, Table S1, and Table S2). Unsupervised hierarchical clustering and principal component analysis confirmed reproducibility between the biological triplicates (Fig. 1, *C* and *D*). A total of 608 (12.9%) unique phosphosites were significantly differentially phosphorylated (Table S3) (<5% false discovery rate (FDR), with a cutoff of ± 1.3 fold change (FC)) relative to the unstimulated control (Fig. 1, *E* and *F* and Table S3). Of these, phosphorylation was significantly increased in 163 phosphosites (from both time points) in response to αCD3 stimulation and in 177 in response to αCD3+PD-L2-Fc ligation, and 95 phosphosites were shared between the two conditions. αCD3 stimulation led to decreased phosphorylation of 13 sites, whereas αCD3+PD-L2-Fc ligation led to significantly decreased phosphorylation of 153 sites. Forty-six sites showed significantly decreased phosphorylation in response to both conditions relative to the unstimulated control (Fig. 1, *G* and *H*). The majority of significantly differentially phosphorylated sites in response to PD-1 ligation were driven by αCD3 stimulation, underling the requirement for TCR signaling prior to PD-1 engagement (Fig. S1G). PD-1 ligation led to decreased phosphorylation of Tyr, Thr, and Ser sites that were phosphorylated by αCD3 stimulation to directly oppose downstream TCR activation. However, PD-1 ligation also enhanced Ser and Thr phosphorylation events >1.3-fold in 53 phosphosites 30 s after stimulation and in 105 sites 5 min after stimulation relative to αCD3 alone. We also detected increased Tyr phosphorylation of four proteins (CCDC110 Tyr-749 and MAPK1 Tyr-187 at 30 s and VPS8 Tyr-1307 and ATP2C2 Tyr-123 at 5 min) downstream of PD-1. PD-1 ligation also led to a greater proportion of decreased Ser, Thr, and Tyr phosphorylation at 30 s compared with αCD3 stimulation alone. By 5 min, the majority of detected Ser and Thr sites in response to PD-1 ligation were up-phosphorylated, whereas the majority of Tyr sites had decreased phosphorylation. Collectively, these data demonstrate that PD-1 signaling results in both increased and decreased phosphorylation of sites that are not restricted to tyrosine residues but also include serine and threonine sites.

### PD-1 signaling engages specific novel biological processes

**Figure 2:**
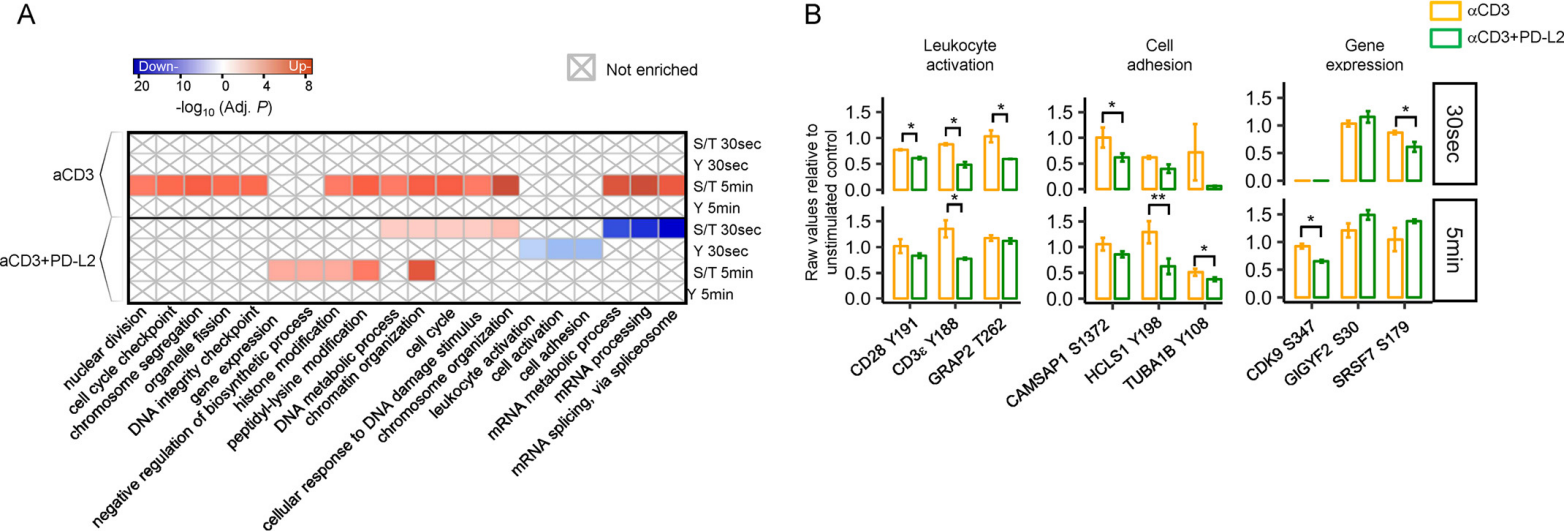
**PD-1–signaling engages specific and novel biological processes.***A*, functional enrichment heatmap of treatment-specific, differentially phosphorylated sites. The color intensity corresponds to the –Log_10_ BH adj. *p*-value for pathways annotated from proteins with increased (*red*) or decreased (*blue*) phosphosites. The × symbol designated pathways that were not enriched. *B*, boxplots of representative differentially phosphorylated phosphosites from the three experimental replicates within each functional category showing raw data relative to the unstimulated control. Bar plots show means ± S.D.

To annotate the PD-1–regulated phosphoproteome into specific biological processes, we performed functional enrichment analysis of treatment-specific, differentially phosphorylated proteins at 30 s and 5 min in response to αCD3 alone or αCD3+PD-L2-Fc (Fig. 2*A*). The majority of down-phosphorylated tyrosine sites in response to PD-1 ligation were confined to proteins specifically involved in three biologic process: leukocyte activation, cell activation, and cell adhesion. For example, PD-1 signaling led to significant down-phosphorylation of CD3ζ and CD3ε ITAMs, the coreceptor CD28, the actin-regulatory protein HCLS1, and tubulin as early as 30 s after ligation (Fig. 2*B*). PD-1 signaling also led to significantly decreased Ser and Thr phosphorylation of proteins required for mRNA processing and splicing at 30 s, followed by an increased phosphorylation of proteins involved in the negative regulation of gene expression and translation at 5 min (Fig. 2*B*). At both time points, we detected phosphorylation of well-known TCR activation-regulated sites in response to αCD3 stimulation within proteins comprising the leukocytes activation group. A smaller number of those detected phosphorylation events passed FDR corrected *p*-value and 1.3 FC and were not sufficient to enrich for that biological process in the αCD3 group, likely because of the absence of CD28 co-stimulation. Nevertheless, PD-1 ligation resulted in highly significant decrease in phosphorylation of key tyrosine residues associated with TCR signaling. Collectively, our functional enrichment analysis revealed that evaluating the decreased phosphorylation of tyrosine phosphosites only within proteins involved in the TCR signaling cascade provides a limited perspective of the pathways engaged by PD-1. In fact, PD-1 signaling led to both increase and decrease in phosphorylation of Ser and Thr sites within proteins involved in leukocyte activation, cellular adhesion, and importantly, gene expression.

### PD-1 attenuates proximal TCR signaling

**Figure 3:**
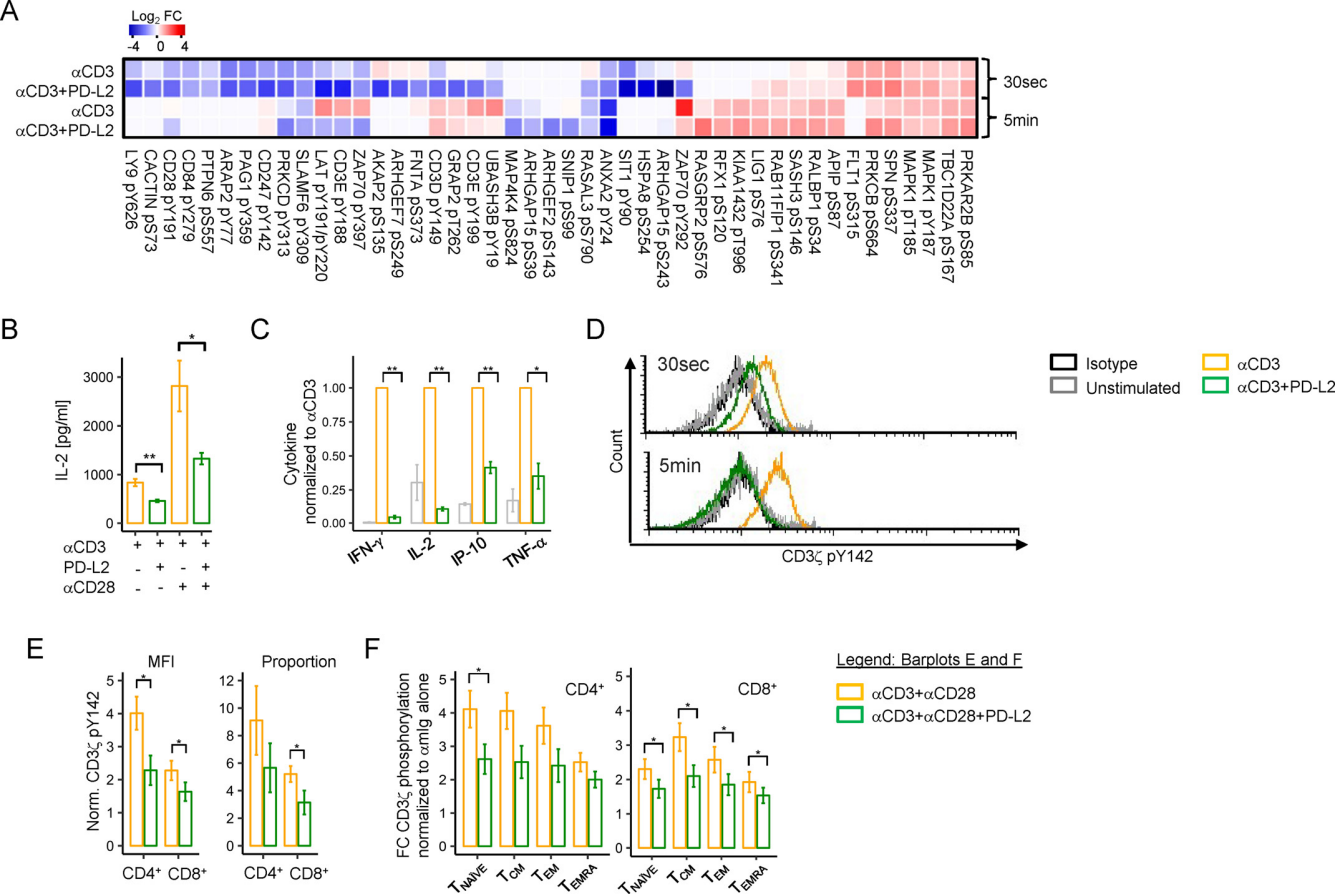
**PD-1 attenuates proximal TCR signaling.***A*, heatmap of TCR signaling phosphosites targeted by PD-1 showing Log2FC values relative to the unstimulated control. *B*, overnight IL-2 secretion assay from Jurkat cells in response to plate-bound αCD3 or plate-bound αCD3 with PD-L2-Fc in the presence or absence of soluble αCD28. *C*, cytokine production from primary human T cells following 48-h stimulation with αCD3 or αCD3+PD-L2 beads from three independent experiments performed in duplicate from two donors. *D*, representative experiment of CD3ζ pTyr-142 phosphorylation of PD-1–transduced Jurkat cells stimulated with beads conjugated to αCD3 or αCD3+PD-L2. *E*, CD3ζ pTyr-142 phosphorylation following ligand crosslinking in primary human CD4+ and CD8+ T cells and in different T cell subsets (*F*) from five independent experiments in three different donors. Data in bar graphs represent mean values ± S.E. (*error bars*). Statistical analyses were performed using paired Student's *t* test, where *p ≤ 0.05 and **p ≤ 0.01.

It is well-known that PD-1 ligation leads to decreased tyrosine phosphorylation of key proteins in the TCR signaling cascade. However, the extent of PD-1–mediated inhibition in proximal TCR signaling has not been evaluated. Our phosphoproteomic data demonstrate that in addition to dephosphorylating the ITAMs of the CD3 complex and pTyr-191 of CD28 as previously reported (11, 18), PD-1 ligation leads to significantly decreased Tyr phosphorylation of key residues within proteins critical for T cell signaling. Among these are LAT pTyr-220, LY9 pTyr-626, PRKCD pTyr-313, ZAP70 pTyr-292 and pTyr-397, PAG1 pTyr-359, and SIT1 pTyr-90 (Fig. 3*A* and Table S3). We also uncovered that PD-1 signaling significantly decreased phosphorylation of pSer residues within GTPase-associated proteins including ARHGAP15 pSer-243 and pSer-39, RASAL3 pSer-790, ARHGEF2 pSer-143, ARHGEF7 pSer-249, and dephosphorylated pThr-262 from GRAP2. Additionally, PD-1 signaling resulted in significantly decreased phosphorylation of multiple cell cycle proteins as early as 30 s, including SKP1 pThr-131, ANP32B pThr-244, and the Ser and Thr phosphatase PP-1A pTyr-306, which likely mediates its inhibitory role in T cell proliferation (Fig. 3*A*, Table S4, and Fig. S2A).

Functionally, PD-1 ligation led to significantly decreased IL-2 production following TCR stimulation (Fig. 3*B*) in Jurkat T cells. CD28 has been previously reported as the primary target for PD-1. Whereas CD28 potentiated the amount of secreted IL-2, the net inhibitory effect of PD-1 on IL-2 production was similar regardless of CD28 co-stimulation (Fig. 3*B*). These findings were replicated in primary human T cells, and 48-h co-culture of T cells with αCD3+PD-L2 beads without CD28 co-stimulation led to significantly decreased secretion of IL-2 and other pro-inflammatory cytokines such as interferon γ (IFN-γ), tumor necrosis factor α, and IP-10 (Fig. 3*C*).

Next, we validated our MS findings by phospho-flow cytometry. First, we confirmed that PD-1 ligation decreased the phosphorylation of the inhibitory Tyr-292 of ZAP70 in Jurkat T cells at 5 min (Fig. S2B). Next, we augmented the sensitivity of our phospho-flow cytometry approach by stably overexpressing PD-1 in Jurkat T cells (Fig. S2B). PD-1 ligation in Jurkat T cells stably expressing PD-1 also led to the dephosphorylation of CD3ζ Tyr-142 ITAM at both 30 s and 5 min after stimulation (Fig. 3*D*). Next, we measured CD3ζ dephosphorylation in primary human CD4+ and CD8+ T cells (Fig. S2D) and confirmed that pTyr-142 is dephosphorylated upon PD-1 ligation (Fig. 3*E*). Following, we also determined CD3ζ pTyr-142 in different memory T cell subsets based on the expression of CD45RA and CCR7 to delineate naïve (CD45RA+CCR7+), effector memory (CD45RA−CCR7−), central memory (CD45RA−CCR7+), and terminally differentiated effector T cells (CD45RA+, CCR7−). Notably, CD3ζ pTyr-142 dephosphorylation was T cell subset–specific (Fig. 3*F*) and independent of surface CD3 or PD-1 expression levels (Fig. S2E). For example, effector memory CD4+ and CD8+ T cells, which express the highest levels of PD-1, were less susceptible to pTyr-142 dephosphorylation compared with naïve and central memory T cells. Additionally, terminally differentiated effector T cells, which had similar levels of PD-1 expression as naïve T cells, were the least susceptible to PD-1–driven pTyr-142 dephosphorylation (Fig. 3*F*).

### PD-1 restricts immune synapse maturation and cellular adhesion

**Figure 4:**
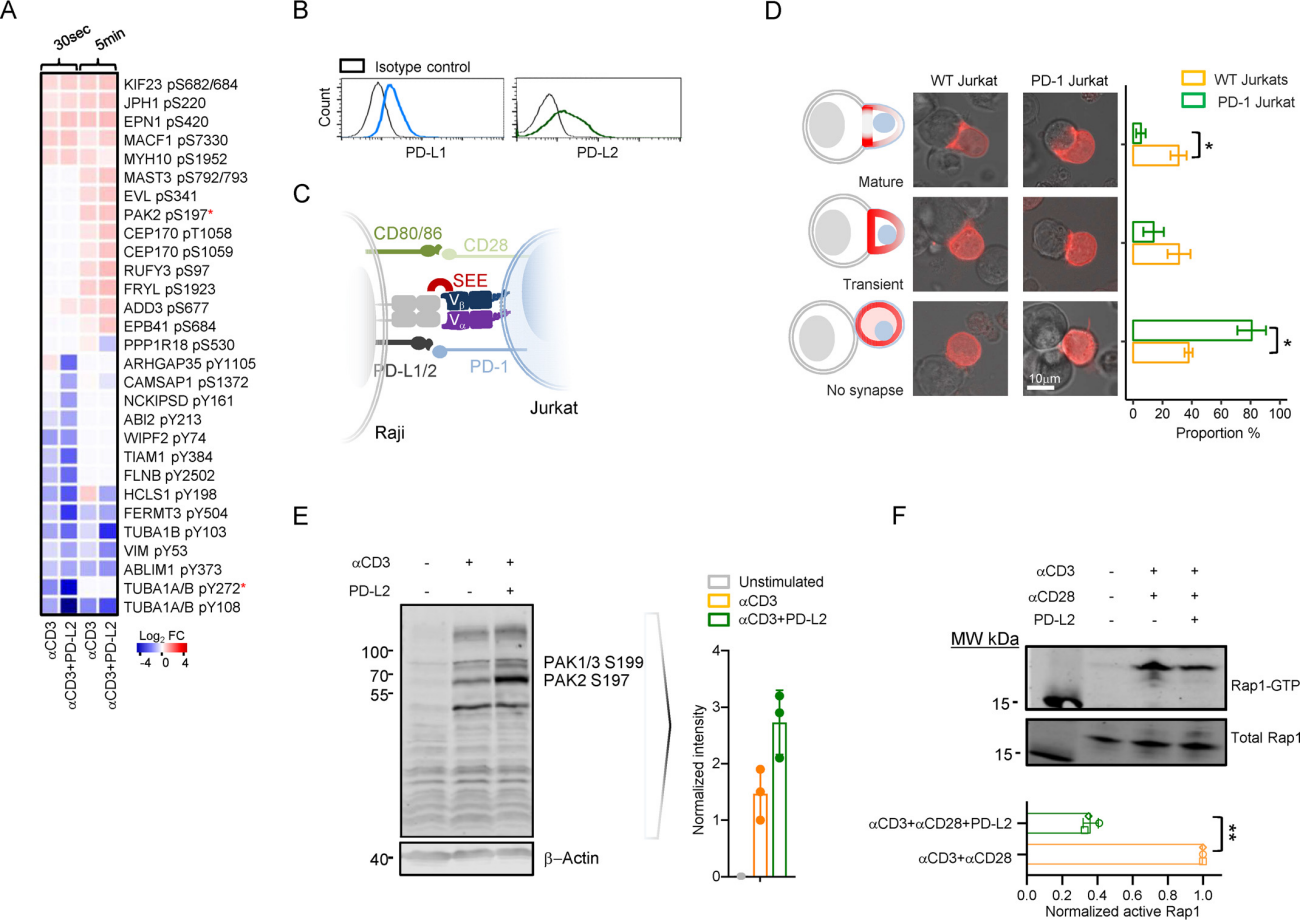
**PD-1 interferes with immune synapse maturation and T cell adhesion.***A*, heatmap of phosphosites within proteins important for cytoskeletal organization targeted by PD-1 showing Log2FC values relative to the unstimulated control. *B*, histogram plots showing surface expression levels of PD-1 ligands in Raji B cells and (*C*) diagram of Raji-Jurkat conjugate assay. *D*, microscopic evaluation of IS formation following SEE-coated Raji cell co-incubation with either WT Jurkat cells, which express low levels of surface PD-1, or PD-1–transduced Jurkat clone. Schematic representation of conjugate formation (*D*, *left*), confocal images of Raji-Jurkat conjugates in parental Jurkat cells, and PD-1–transduced Jurkat clone (*D*, *center*), transfected with LifeAct, and bar graph of mean ± S.E. (*error bars*) of the quantitated proportion of conjugate formation in each group (*D*, *right*) from three independent experiments. *E*, Western blotting analysis and quantitation of PAK2 Ser-197 phosphorylation from *n* = 3 independent experiments in Jurkat T cells after 5-min stimulation with Dynabeads coated with either αCD3 alone or αCD3+PD-L2. *F*, a representative blot of active GTP-bound Rap1 pulldown assay following 5-min crosslinking stimulation in WT Jurkat T cells from three independent experiments (bar graph shows mean ± S.D. (*error bars*)). Statistical analysis was performed using unpaired (*D*) or paired (*F*) Student's *t* test, where *p ≤ 0.05 and **p ≤ 0.01.

Previous studies have reported that PD-1 impairs T cell adhesion (12, 19). However, the cytoskeletal proteins that are engaged by PD-1 signaling, which ultimately leads to impaired cytoskeletal protein dynamics, have not been documented before. Our phosphoproteomic analysis demonstrated that PD-1 ligation decreased the phosphorylation of tyrosine residues from key proteins involved in cytoskeletal organization and immune synapse (IS) formation (Fig. 4*A* and Table S4). Among those were the actin-regulatory proteins NCKIPSD pTyr-161 and ABI2 pTyr-213, the IS actin-regulatory adaptor HCLS1 pTyr-198, and tubulin pTyr-272 (20, 21, 22). The majority of these tyrosines were significantly dephosphorylated at 30 s of T cell stimulation in the presence of PD-1 ligation. Thus, we reasoned that PD-1 ligation interferes with IS formation by directly targeting actin dynamics and synapse maturation. To test this, we transfected parental Jurkat T cells and Jurkat T cells stably expressing high levels of human PD-1 with mCherry-LifeAct, which is a 17–amino acid peptide that stains filamentous actin and allows *in vivo* visualization of actin dynamics. Immune synapse formation results in early recruitment of actin to the center of the synapse, which indicates the initial phase of synapse formation (23). Shortly after that, the actin redistributes to the periphery of the synapse while being depleted from the center. This is the phase of synapse maturation. We cocultured *Staphylococcus* enterotoxin E (SEE)-loaded Raji B cells, which express moderate and stable levels of both PD-L1 and PD-L2 (Fig. 4*B*) and MHC class II proteins, with either parental Jurkat T cells or Jurkat T cells stably expressing high levels of human PD-1 (Fig. 4*C*) and recorded the actin localization visualized by the LifeAct probe. SEE forms a trimolecular complex with MHC class II expressed on the surface of Raji B cells with the Vβ chain of the TCR, thus allowing for conjugate formation. Our results show that PD-1–expressing Jurkat T cells were unable to form stable mature synapses with Raji B cells (Fig. 4*D*), demonstrating that PD-1 ligation also inhibits IS formation by targeting key proteins of the actin cytoskeleton.

We identified that PD-1 ligation in WT Jurkat T cells led to significantly increased phosphorylation of P21-activated kinase 2 (PAK2) at Ser-197 at 5 min after stimulation (Fig. 4*A*). As a direct effector of the Rho family GTPases Rac and Cdc42, PAK2 regulates actin cytoskeletal remodeling. At its basal state, PAK2 is autophosphorylated at five different serine sites, including Ser-197 detected in our study. We also validated these findings by Western blotting and confirmed that PD-1 ligation increased the phosphorylation of Ser-197 in PAK2 (Fig. 4*E*). Collectively, the differential PD-1 driven phosphorylation of key proteins regulating cytoskeletal organization and synapse formation led us to determine whether PD-1 ligation interfered with cellular adhesion. When we measured the levels of active GTP-loaded Rap1 protein, a key regulator of cellular adhesion and synapse formation, PD-1 ligation led to decreased levels of GTP-loaded Rap1 (Fig. 4*F*).

### PD-1 represses both gene expression and protein translation

**Figure 5:**
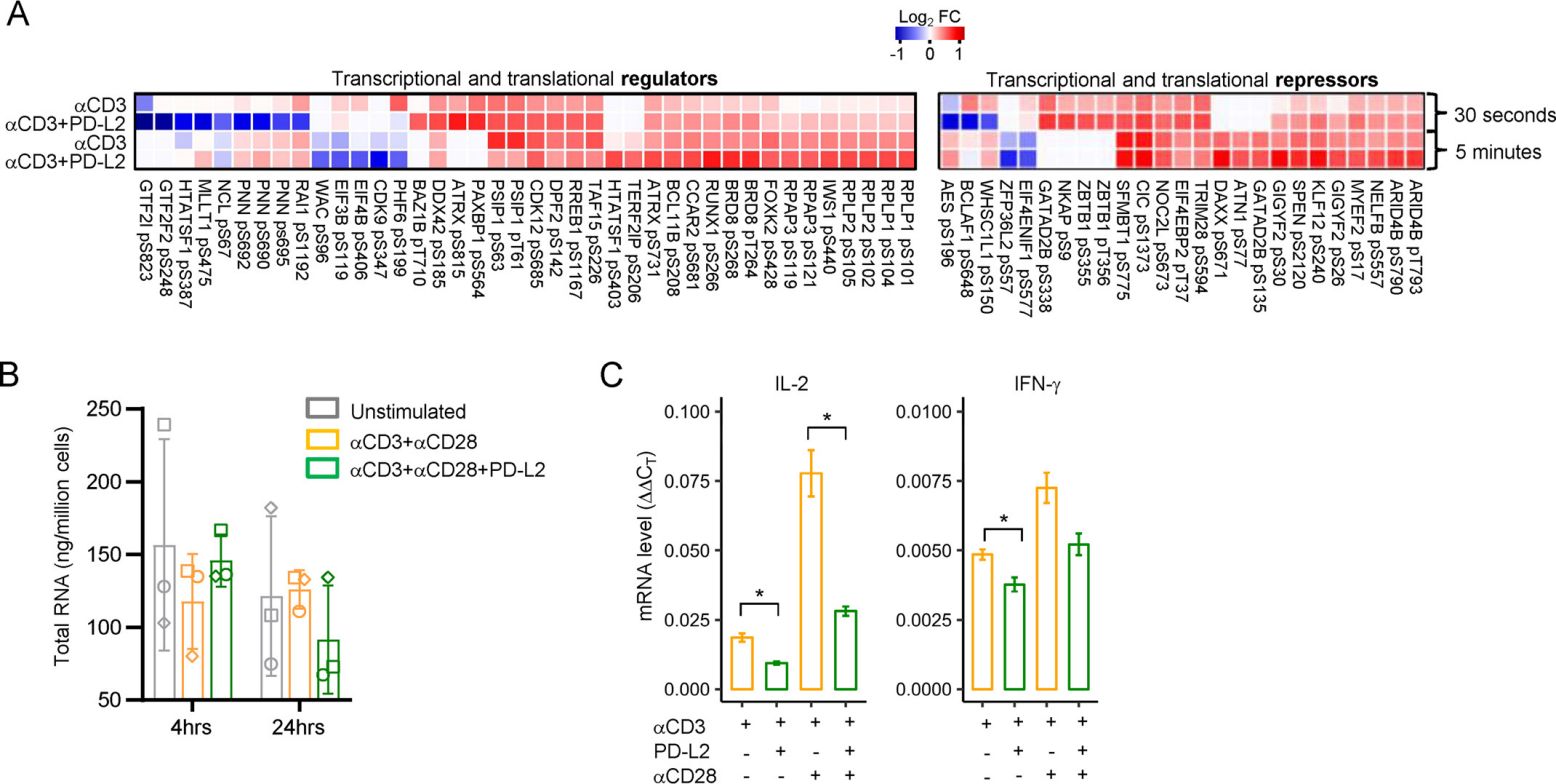
**PD-1 ligation changes the Ser/Thr phosphorylation landscape of both transcriptional and translational regulators and repressors.***A*, heatmaps of Log2FC of normalized phosphosite intensities relative to the unstimulated control for transcriptional and translational regulators and repressors. *B*, total RNA production from live Jurkat cells stimulated at the indicated time points with either plate-bound αCD3 or αCD3+PD-L2-Fc in presence of soluble αCD28. The bar graphs show mean ± S.D. (*error bars*) of *n* = 3 independent experiments. *C*, RT-qPCR analysis of IL-2 and IFN-γ expression from Jurkat cells following 24-h plate-bound stimulation as in (*B*) in the presence or absence of soluble αCD28 and *p ≤ 0.05.

The early consequences of PD-1 ligation also led to the differential phosphorylation of proteins involved in the negative regulation of gene expression and protein translation. In fact, the greatest proportion of differentially phosphorylated serine and threonine residues was in proteins associated with mRNA processing and splicing (Fig. S3), transcriptional and translational regulation (Fig. 5*A*, *left panel*), and repressors of gene expression and translation (Fig. 5*A*, *right panel* and Table S4). PD-1 ligation led to significant dephosphorylation of Ser and Thr residues at multiple sites within proteins associated with spliceosome assembly, such as SRRM1, SRRM2, SF1, SRSF3, and SRSF4 (Fig. S3). More notably, PD-1 ligation led to the significant dephosphorylation of CDK9 pSer-347 and the translation initiation factors EIF3B pSer-119 and EIF4B pSer-406 after 5 min of stimulation (Fig. 5*A*).

These striking observations led us to determine whether PD-1–mediated inhibition of cytokine production was due to transcriptional or translational repression, or both. We determined the amount of total RNA production following PD-1 ligation at 4 and 24 h post-stimulation in Jurkat T cells. There was a decreased amount of total RNA produced by the cells in the presence of PD-1 ligation (Fig. 5*B*) after 24 h of stimulation. Moreover, PD-1 inhibited IL-2 and INF-γ secretion and their mRNA levels as determined by RT-qPCR (Fig. 5*C*). Importantly, the inhibitory potential of PD-1 was observed regardless of CD28 co-stimulation. These results suggest that PD-1 inhibits T cell gene expression and protein translation by interfering with both RNA processing and protein translation.

### Prediction analysis of PD-1–regulated kinase-substrate relationships

**Figure 6:**
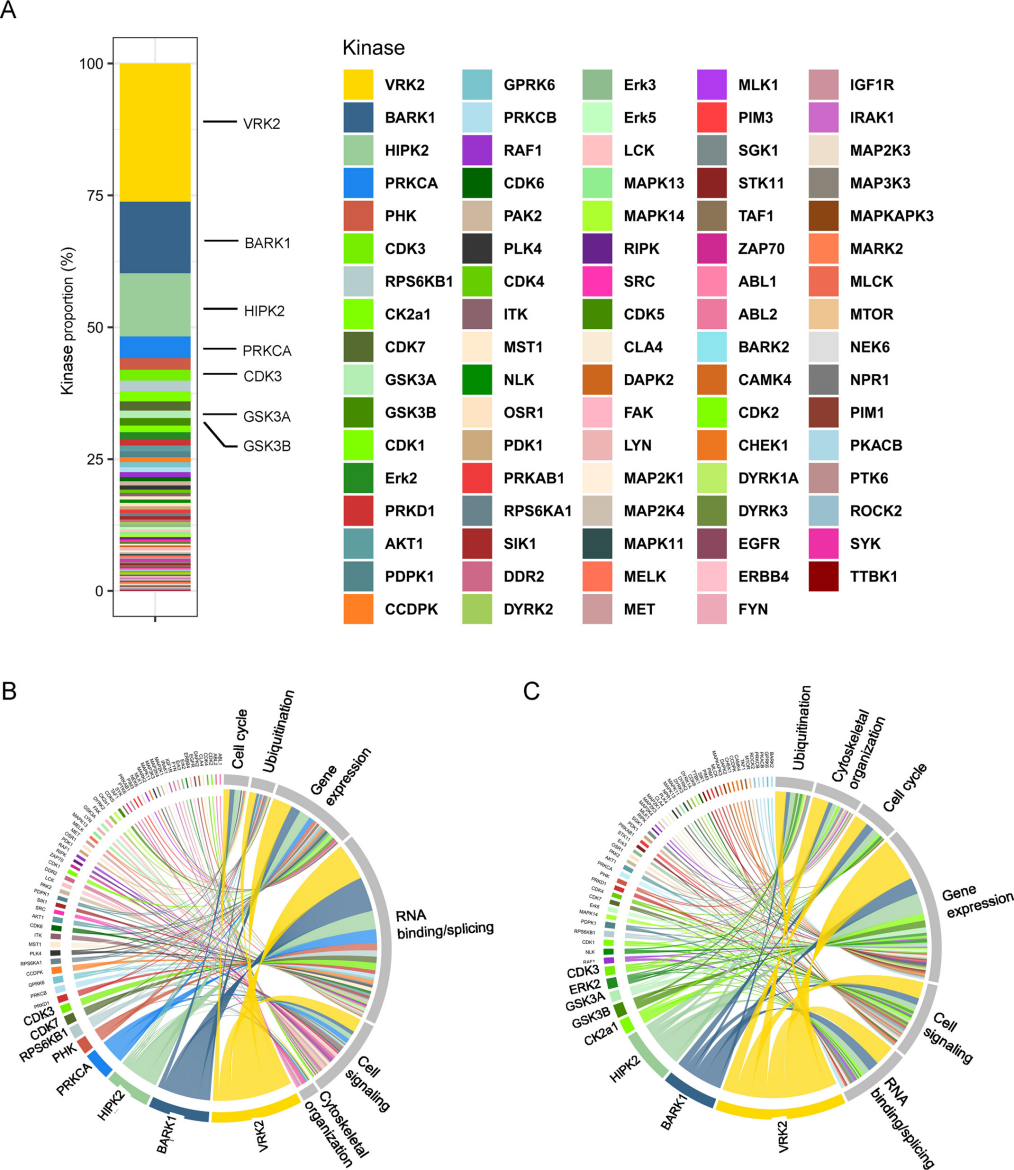
***In silico* prediction analysis of kinase/substrate interactions downstream of PD-1.***A*, proportion of phosphosites targeted by each kinase. Chord diagrams showing the distribution of kinases predicted to phosphorylate sites with decreased (*B*) and increased (*C*) phosphorylation following PD-1 ligation according to the functional group of each protein containing that phosphosite.

We sought to determine what were the kinases that phosphorylated PD-1 targeted phosphosites and whose downstream signaling may be disrupted by PD-1 blockade. We carried out motif prediction analysis and database search for known substrates to determine which kinases phosphorylated the Tyr, Ser, and Thr residues within our functional groups that were significantly differentially phosphorylated following PD-1 ligation. These *in silico* analyses identified 84 kinases (Table S5). Of those, the serine/threonine kinases vaccinia related kinase 2 (VRK2), β-adrenergic receptor kinase 1 (BARK1), homeodomain-interacting protein kinase 2 (HIPK2), and protein kinase C isozyme α (PRKCA) were predicted to phosphorylate ∼50% of all differentially phosphorylated Ser and Thr substrates downstream of PD-1 (Fig. 6*A*). These kinases primarily phosphorylated proteins involved in RNA binding, splicing, and gene expression for both Ser and Thr phosphosites that both decreased (Fig. 6*B*) and increased (Fig. 6*C*) in phosphorylation following PD-1 ligation. Collectively, our findings reveal effector kinases downstream of PD-1 signaling that may be actionable targets for novel therapies, and their involvement in the PD-1 pathways warrants further investigation.

## Discussion

Reinvigorating T cell–mediated anti-tumor immunity is a prerequisite for the development of successful immunotherapies (24, 25). Therapeutic blockade of PD-1, or its ligand PD-L1, has proven successful for a number of cancer indications. However, not all patients have durable responses to these therapies, and a significant proportion of patients experiences immune toxicity, emphasizing the critical role of PD-1 in immune tolerance (26, 27). To develop more successful therapeutics targeting the PD-1/PD-L1 axis, it is imperative that we understand the underlying molecular mechanism and signaling pathways engaged by PD-1. Here, we carried an unbiased phosphoproteomic screen of PD-1–triggered pathways following ligation with PD-L2 in Jurkat T cells. We chose this system because Jurkat T cells offer a high degree of populational and functional uniformity. Our global phosphoproteomic screen reveals multiple molecular tiers of PD-1–engaged signaling pathways, extending beyond the known TCR proximal signaling events. These molecular networks converged into three main specific functional groups encompassing leukocyte activation, cellular adhesion, and control of gene expression, ultimately leading to attenuated T cell function. We validated these functional PD-1 substrates with various *in vitro* immunoassays. Importantly, based on our phosphoproteomic findings we were able to predict kinases that are potential downstream effectors of PD-1 signaling (28, 29).

Following TCR ligation with peptide-MHC, PD-1 binds its ligands PD-L1 and PD-L2 and localizes within the IS (8). To execute its function, PD-1 recruits the tyrosine phosphatase SHP-2 to its cytoplasmic domain, which then dephosphorylates tyrosine residues within proteins in the proximity of the IS. As a result, several proteins within the TCR signaling cascade have been demonstrated as substrates for SHP-2–mediated dephosphorylation following PD-1 ligation (11, 12, 18). Here we confirmed that Tyr-188 and Tyr-198 ITAMs of CD3ε and Tyr-142 of CD3ζ, and Tyr-191 of CD28, are key targets for dephosphorylation following PD-1 ligation. We also confirmed that CD3ζ pTyr-142 is targeted by PD-1 in *in vitro* signaling studies of both Jurkat and primary human CD4+ and CD8+ T cells. Interestingly, the surface expression levels of PD-1 in different T cell subsets did not correlate with its propensity to dephosphorylate this ITAM, suggesting that PD-1 may exert T cell subset–specific functions. Our studies extend the list of tyrosine phosphosites targeted by PD-1 to the membrane adaptor proteins LAT pTyr-191, SIT1 pTyr-90, and PAG1 pTyr-359 (30). LAT pTyr-191 is phosphorylated by ZAP70 and serves as a high-affinity site for GADS SH2 binding and initiates the signaling cascade coupling the TCR to distal intracellular events, leading to T cell activation (31, 32). Decreased LAT Tyr-191 phosphorylation will interfere with SLP76 recruitment and subsequent binding of PLCγ1 leading to decreased calcium flux and nuclear factor of activated T cells (NFAT) translocation, eventually causing inhibition of IL-2 production. Collectively, decreased CD3ζ pTyr-142 causes less recruitment of ZAP70. Subsequently, decreased LAT pTyr-191 phosphorylation recruits less PLCγ1, which leads to decreased calcium flux and nuclear NFAT translocation, eventually causing inhibition of IL-2 production.

Other proteins targeted by PD-1 included PRKCD pTyr-313, which is essential for tolerance and homeostasis (33). In addition, PD-1 ligation led to dephosphorylation of the SLAM family members LY9 (SLAMF3), CD84 (SLAMF5), and SLAMF6, which are implicated in T cell activation by recruiting the adaptor protein SH2D1A/SAP via tyrosine-based interactions with its SH2 domain (34).

Through this work, we cannot exclude the possibility that some of the events downstream of PD-1 are indirectly mediated by inhibiting LCK and ZAP70. On the one hand, we show that the activating Tyr-394 of LCK was targeted to a greater extent by PD-1 ligation, suggesting that some of the tyrosine dephosphorylated sites downstream of this receptor are due to down-modulated LCK activity. On the other hand, a previous report demonstrated that although PD-1 targeted both the activating (Tyr-394) and the inhibitory (Tyr-505) tyrosines of LCK, it had a net-positive effect on LCK activity (18). Similarly, we observed that PD-1 primarily targeted the inhibitory Tyr-292 of ZAP70, suggesting that although PD-1 targets LCK and ZAP70, its inhibitory functions are not mediated solely by inhibiting these kinases, and further studies are needed to completely uncover these relationships.

Hui *et al*. (18) have previous reported that CD28 is a target of PD-1 inhibition. Although the phosphoproteomic data support that, our functional experiments provide evidence that PD-1 is able to exert its inhibitory function in the absence of CD28 co-stimulation. Although CD28 ligation resulted in greater response magnitude, overall PD-1 inhibition of T cell responses was similar in the presence or absence of CD28 co-stimulation, suggesting that CD28 is not the sole determinant of PD-1-mediated T cell inhibition (35). This is not surprising because PD-1 blockade successfully re-invigorates anti-tumor T cell responses, yet the majority of tumors do not express B7.1 or B7.2 proteins, the ligands for CD28. Nevertheless, PD-1–mediated inhibition of CD28 co-stimulation may attenuate anti-cancer immune responses at sites of immune priming, a critical step in initiating anti-tumor immunity. Our study complements previous findings of the importance of co-stimulation in PD-1 inhibition (36) and sheds light into the pathways triggered by PD-1 directly downstream of the TCR, a context highly relevant to T cell responses in the tumor microenvironment. It is likely that CD28 dependence is context-dependent and not the sole determining factor of the inhibitory function of PD-1 in T cells. Further molecular and functional studies in primary human T cells are required to dissect the contribution of CD28 co-stimulation to PD-1 signaling.

Remarkably, our phosphoproteomic approach uncovered that PD-1 also targeted tyrosine residues within proteins that are critical for cytoskeletal organization, cellular adhesion, and immune synapse formation. These processes regulate T cell mobility, polarization, IS formation, and proliferation and are essential for protective T cell responses. We corroborated these data with *in vitro* functional assays and demonstrated that these early events translated into PD-1–mediated inhibition of T cell adhesion, IS formation, and T cell proliferation. By revealing the phosphoproteomic networks involved in PD-1 regulation of T cell adhesion and cytoskeletal organization, our data provide mechanistic basis for PD-1 interference with T cell motility (37, 38). Collectively, these results point to PD-1–regulated fine stoichiometric balance of activating tyrosine phosphorylation within the TCR signaling cascade that needs to be achieved for propagation of proximal TCR signaling and downstream T cell activation.

We also identified a significant proportion of dephosphorylated Ser and Thr sites within proteins distal to the TCR and confined to cellular gene expression, mRNA splicing, and initiation of protein translation. This is likely indirectly mediated by PD-1/SHP-2. Although tyrosines are direct targets for PD-1/SHP-2, down-modulation of activating tyrosines in serine/threonine kinases, such as PRKCD, may result in decreased Ser and Thr phosphorylation. In contrast, targeting tyrosines in serine/threonine phosphatases will result in increased Ser and Thr phosphorylation. For instance, pTyr-306 of PPP1CA was significantly dephosphorylated following PD-1 ligation. The majority of phosphosites with significantly increased phosphorylation were confined to serine residues within protein repressors of gene expression and translation. How PD-1 interferes with mRNA transcription and protein translation requires further investigation to determine whether this is a global PD-1–mediated effect or restricted to a given group of signaling mediators.

To give a translational application to our findings and in an attempt to look for potential complementary treatments to PD-1 blockade, we determined the putative kinases that might phosphorylate PD-1–targeted phosphosites and confirmed their function *in vitro*. We identified that the serine/threonine kinases VRK2, HIPK2, BARK1, and PRKCA phosphorylated ∼50% of the Ser/Thr sites implicated in PD-1 signaling. Although our prediction algorithm has limitations, these data demonstrate the involvement of multiple kinases in PD-1 signaling. Further studies are needed to functionally validate putative kinases that may mediate signaling downstream of PD-1 and serve as potential therapeutic targets. These findings expand upon our current view of PD-1 signaling and show that PD-1 immunoregulation extends beyond dephosphorylation of tyrosine residues. Our data together with recent findings showing that SHP-2 is dispensable for PD-1 signaling (39) suggest that PD-1 may also engage additional phosphatases to mediate its inhibitory function. However, unlike kinases, the ubiquitous enzymatic specificity and function of phosphatases creates a challenge for motif prediction analysis.

Our reductionist approach has some limitations. First, there are differences in the phosphorylation magnitude between primary human T cells and Jurkat T cells (40). To this end, we validated some of the conserved TCR signaling pathways targeted by PD-1 in both Jurkat and primary CD4+ and CD8+ T cells. Second, given that cancer-specific memory CD8+ T cells represent a primary target for cancer immunotherapy and that CD4+ T cells play a critical “helper” role in the execution of CD8+ responses, it is of great importance to identify T cell subset–specific differences in PD-1 signaling and its related molecular pathways. Third, Jurkat T cells have constitutive PI3K signaling because they lack phosphatase and tensin homolog (PTEN). Although constitutive PI3K signaling in Jurkat T cells may affect downstream molecular responses, it does not preclude the validity of our findings with regard to proximal TCR signaling, synapse formation, and gene expression regulation. A substantial portion of the PD-1–regulated phosphoproteome is membrane proximal and tyrosine-dependent because of the anatomical location of this receptor and its recruitment of SHP2. In fact, Vale *et al*. (18) and our study show that PD-1 targets pTyr-191 of CD28, which is a binding site for p85 subunit of PI3K (41). Earlier reports have also shown that p85 binds to CD3 ITAMs and that PD-1 ligation led to decreased phosphorylation of CD3ε, CD3ζ, and CD3γ ITAMs. Therefore, whereas Jurkats have constitutive PI3K signaling, PD-1 ligation likely disrupts PI3K recruitment by dephosphorylating tyrosine residues within the PI3K recognition motif (PI3K binds to two closely spaced pY*XX*M motifs). Consequently, constitutive PI3K signaling is unlikely to alter PD-1–targeted phosphosites and conserved T cell responses that we examine in this study. Given the critical role of CD28 in T cell co-stimulation and previous studies implicating it as a major target for PD-1 signaling (18, 36), it is important to investigate its contribution to PD-1–mediated immunoregulation. Finally, our prediction algorithm (Fig. 6) relied on publicly available kinase-substrate datasets. These results are largely dependent on the availability of an exhaustive training dataset validated for each kinase that will allow accurate prediction of the kinase motif. Further investigations are required to confirm the role of the most prominent kinases in PD-1 biology. We hope that the current study serves as a springboard for future investigations of PD-1 signaling in antigen-specific primary CD4+ and CD8+ T cell subsets, which may help identify novel anti-cancer therapeutics.

In conclusion, our global phosphoproteomic approach offers a comprehensive resource into the signaling pathways triggered by PD-1. Our analysis revealed that in addition to key activating tyrosines within proteins initiating the TCR signaling cascade, PD-1 signaling resulted in dephosphorylated tyrosine residues within proteins mediating IS formation, cellular adhesion, and cytoskeletal organization. Interestingly, PD-1 also engaged pathways involved in the repression of gene expression and protein translation. Whether these pathways are engaged in a T cell subset–specific manner remains to be further investigated. Nevertheless, dissecting PD-1 signaling networks is key to understanding its function as a critical immune-regulatory receptor and for the development of improved therapeutic approaches.

## Experimental procedures

### Study design

To dissect the signaling pathways downstream of PD-1, we used Jurkat E6.1 T cell line and TMT-based quantitative LC–MS/MS to obtain an unbiased view of the PD-1–regulated phosphoproteome. We applied computational approaches to our phosphoproteomic data to define the signaling pathways engaged by PD-1. To confirm the functional consequences of our phosphoproteomic results, we carried out *in vitro* controlled laboratory experiments in Jurkat T cells and primary human T cells and employed conventional and phospho-flow cytometry, microscopy, ELISA, T cell proliferation, immunoblot, and molecular cloning. The number of samples, statistical tests, and experimental replicates are designated in the Fig. legends. The study was approved by the Institutional Review Board at Columbia University Medical Center and all donors provided informed consent.

### Preparation of ligand-coupled beads

Dynabeads M270-Epoxy (Thermo Fisher Scientific) were covalently conjugated with combinations of mouse anti-human CD3 antibody (clone UCHT1, BioLegend), recombinant human PD-L2-human IgG1 Fc chimera protein (R&D Systems), or mouse IgG1 isotype antibodies (R&D Systems) following the manufacturer's recommendations. The molar ratio between anti-human CD3 and PD-L2 Fc was 1:3, and the molar ratios of all added proteins were kept constant by the addition of mouse IgG isotype antibodies. For each preparation, we used 8 μg of total protein/1 mg of beads, of which 2 μg were anti-CD3 and 4 μg were PD-L2 Fc.

### Sample preparation

100 × 10^6^ Jurkat T cells were activated in three biological replicates with anti-human CD3–, anti-human CD3+PD-L2 Fc–, or IgG isotype–coated beads at a ratio of 1:1 for either 30 s or 5 min followed by washing with ice-cold PBS and lysis with urea lysis buffer, according to the P-Tyr-1000 kit protocol (Cell Signaling Technology). Lysates were reduced with 5 mm DTT (Sigma-Aldrich) at 57 °C for 60 min and alkylated with 10 mm iodoacetamide (Sigma-Aldrich) for 60 min in the dark. Proteins from each sample were then digested overnight at 37 °C using sequencing grade trypsin (1:40) (Promega). Tryptic peptides were labeled with 10-plex isobaric TMT (Thermo Fisher Scientific) according to manufacturer's instructions. Samples from the different time points were labeled with different TMT labeling reagents. The labeling reaction was carried out for 1 h at room temperature and quenched with 5% hydroxylamine (Thermo Fisher Scientific). The digested and labeled peptides from the different time points were pooled and desalted with 5 mg of C18 SEP-PAK light reversed phase column (Waters), followed by lyophilization and immunoprecipitation according to P-Tyr-1000 kit protocol. Two additional phosphotyrosine immunoprecipitations were performed with anti pTyr-99 (source) and anti-4G10 (Millipore) antibodies utilizing the flow-through. Next, titanium dioxide enrichment was performed on the flow-through of the phosphotyrosine immunoprecipitation.

#### TMT labeling

The dried peptide mixture was resuspended in 100 mm triethylammonium bicarbonate, pH 8.5, using a volume of 138 μl. Each sample was labeled with TMT reagent according to the manufacturer's protocol. In brief, each TMT reagent vial (0.8 mg) was dissolved in 256 μl of anhydrous ethanol and was added to each sample. The reaction was allowed to proceed for 60 min at room temperature and then quenched using 24 μl of 5% w/v hydroxylamine. The samples were combined at a 1:1 ratio and the pooled sample was subsequently desalted using SCX and SAX solid-phase extraction columns (Strata, Phenomenex).

#### Global proteome analysis

A 500-μg aliquot of pooled sample was fractionated using basic pH reverse-phase HPLC (as described) (42). Briefly, the sample was loaded onto a 4.6 × 250-mm Xbridge C18 column (Waters, 3.5-μm bead size) using an Agilent 1260 Infinity Bio-inert HPLC and separated over a 70-min linear gradient from 10 to 50% solvent B at a flow rate of 0.5 ml/min (buffer A = 10 mm ammonium formate, pH 10.0; buffer B = 90% acetonitrile and 10 mm ammonium formate, pH 10.0). A total of 130 fractions were collected throughout the gradient. The early-, middle-, and late-eluting fractions were concatenated and combined into 40 final fractions. The combined fractions were concentrated in the SpeedVac and stored at −80 °C until further analysis.

#### Phosphopeptide enrichment

A 25-mg aliquot of the TMT-labeled and pooled sample was subjected to three sequential enrichments for tyrosine phosphopeptide using three different antibodies as per the manufacturer's recommended protocols. The antibodies used were p-Tyr-1000 (Cell Signaling Technology), pTyr-99 (Santa Cruz Biotechnology), and 4G10 (EMD Millipore). The flow-through of the pTyr enrichment was subsequently further enriched for phosphopeptides using 5-μm Titansphere^TM^ TiO2 beads (GL Sciences, Torrance, CA) as described (43). Briefly, peptides were reconstituted in binding buffer (1 mm monopotassium phosphate in 65% acetonitrile and 2% TFA). The TiO2 beads were washed three times with washing buffer (65% acetonitrile and 0.1% TFA), added to the peptides at a ratio of 1:4 (peptides to beads), and incubated for 20 min. The flow-through was collected and incubated for 20 min with a fresh aliquot of washed beads. Both sets of beads were washed twice with washing buffer and then loaded onto a C18 STAGE tip where the beads were washed two additional times using washing buffer. Peptides were eluted with 15% ammonium hydroxide in 40% acetonitrile, concentrated in a SpeedVac concentrator, and stored at −80 °C.

#### LC–MS/MS analysis

An aliquot of each sample was loaded onto a trap column (Acclaim® PepMap 100 pre-column, 75 μm × 2 cm, C18, 3 μm, 100 Å, Thermo Fisher Scientific) connected to an analytical column (EASY-Spray column, 50 m × 75 μm ID, PepMap RSLC C18, 2 μm, 100 Å, Thermo Fisher Scientific) using the autosampler of an Easy nLC 1000 (Thermo Fisher Scientific) with solvent A consisting of 2% acetonitrile in 0.5% acetic acid and solvent B consisting of 80% acetonitrile in 0.5% acetic acid. The peptide mixture was gradient eluted into the QExactive mass spectrometer (Thermo Fisher Scientific) using the following gradient: a 2–20% solvent B in 120 min and 20–30% solvent B in 20 min, followed by 30–100% solvent B in 20 min. The full scan was acquired with a resolution of 70,000 (at *m*/*z* 200), a target value of 1e6, and a maximum ion time of 120 ms. After each full scan 10 higher-energy C-trap dissociation (HCD) MS/MS scans were acquired using the following parameters: resolution 35,000 (at *m*/*z* 200), isolation window of 1.5 *m*/*z*, target value of 1e5, maximum ion time of 250 ms, normalized collision energy of 30, and dynamic exclusion of 30 s. An aliquot of the TiO2 phospho-enriched sample was subjected to the same LC and MS instrument acquisition parameters except the gradient was as follows: from 2–20% solvent B in 120 min and from 20–30% solvent B in 50 min, followed by 30–100% solvent B in 20 min, and after each full scan 15 higher-energy C-trap dissociation MS/MS scans were acquired with a normalized collision energy of 27.

#### Data analysis

The raw data were searched using MaxQuant4 version 1.5.2.8. Proteins and peptides were searched against the UniProt human database using the Andromeda search engine (44) integrated within the MaxQuant software suite using a target-decoy approach and the following settings: two missed cleavages were allowed for trypsin; oxidation of methionine, phosphorylation of serine, threonine, and tyrosine, and deamidation of asparagine and glutamine were set as variable modifications; carbamidomethyl of cysteine was set as fixed modifications; and both precursor and fragment mass tolerances were set to 10 ppm. The peptide identifications were filtered using an FDR of 0.01 at both the protein and peptide level. Only unique peptides were used for quantification, and proteins identified with fewer than two unique peptides were excluded from analysis at the global protein level.

### Bioinformatic analysis

Identified phosphopeptides were included only if present in all three replicates with phosphosite localization probability score >0.7 of the Andromeda peptide search engine. Global normalization of reporter ion intensity values was performed relative to the overall abundance of total protein in each replicate to account for differences in initial protein concentration, and the normalized values were log_2_ transformed. Downstream bioinformatic analysis was performed in the R programming environment (RRID:SCR_001905). Differentially regulated phosphosites were determined with a moderated *t* test using the limma package (45) in R with ≤5% false discovery rate and FC of ± 1.3. We used the following R packages for graphical representation of our results: heatmaps were created with gplots; ggplot2 was used for box plots, bar plots, and scatter plots; treemap package was used for the generation of kinase proportions; and chordDiagram was used for circular visualization of kinase/substrate relationships. Phosphorylated residues were extracted and annotated from the PhosphoSite phosphorylation site dataset, and kinase-substrate predictions were identified with GPS3.0 (46) with high threshold setting corresponding to 2% false positive rate for serine/threonine kinases, 4% false positive rate for tyrosine kinases, and an estimated ratio between the prediction score and cutoff value ≥ 2. Known phosphorylation sites were curated manually from the PhosphoSite kinase substrates dataset and the literature.

### Primary human T cell isolation and culture

Peripheral blood was collected from healthy adult donors following informed consent. Total CD3+ T cells were isolated by density gradient centrifugation (Lymphoprep, Axis-Shield PoC AS) and negative selection using the RosetteSep human T cell enrichment mixture (Stemcell Technologies). For CD4+ or CD8+ T cell magnetic separation, CD3+ T cells were labeled with MACS MicroBeads (Miltenyi Biotec) and isolated by positive selection. Primary T cells were either directly used in stimulation assays or expanded *in vitro*. *In vitro* T cell cultures were maintained in complete RPMI, containing 10% FCS, minimum Eagle's medium nonessential amino acids, 1 mm sodium pyruvate, 100 IU/ml penicillin, 100 μg/ml streptomycin, and GlutaMAX-I. For T cell expansion, the media were supplemented with 20 IU/ml recombinant human IL-2 (PeproTech) and 0.5 μg/ml lectin from *Phaseolus vulgaris* (Sigma-Aldrich).

### T cell stimulations

#### Ligand-coated Dynabeads

Dynabeads stimulations for phospho-flow cytometry or Western blotting in Jurkat T cells were performed at a 1:5 cell-to-bead ratio. Resting Jurkats were washed once with PBS at 450 × *g* and room temperature (RT) and resuspended in PBS to a final concentration of 5 × 10^7^ cell/ml. When anti-human CD28 antibody (clone CD28.2, BioLegend) was added, the cells were pre-incubated with the antibody for 10 min at room temperature. For each stimulation, 100 μl of cells were pipetted in Eppendorf tubes and stimulated with 2.5 × 10^7^ beads. The cell/bead mixture was gently resuspended by pipetting, and contact was initiated by brief 15-s centrifugation at 200 × *g* and RT. Next, the suspension was placed at a 37 °C water bath for the indicated time points. Immediately after stimulation, the tubes were processed for either Western blotting or phospho-flow cytometry analysis as described below.

#### Plate-bound ligand

For plate-bound stimulations, 48-well plates were coated overnight at 4 °C with 200 μl of cold PBS containing 10 μg/ml anti-human CD3 with or without 5 μg/ml recombinant human PD-L2 mouse IgG1 Fc chimera protein (ACROBiosystems). On the next day, the wells were washed gently once with cold PBS and the surface was blocked for 10 min with 250 μl of complete warm RPMI. T cells were washed and resuspended to a final concentration of 2 million cells/ml in warm complete RPMI, containing 1 μg/ml anti-human CD28. Finally, 250 μl of cells (5 × 105 cells) was pipetted in each well and the plate was incubated for 18 h at 37 °C, 5% (v/v) CO_2_. IL-2 concentration in the supernatant was measured by ELISA from BioLegend.

#### Ligand crosslinking

For antibody crosslinking stimulations, 5 × 10^5^ CD3+ T cells in 100 μl of cold PBS were incubated for 20 min on ice with soluble anti-human CD3 (10 μg/ml), anti-human CD28 (10 μg/ml), and recombinant human PD-L2 mouse IgG1 Fc (10 μg/ml). The cells were washed once with cold PBS at 450 × *g* and 4 °C and resuspended in cold PBS containing crosslinking goat anti-mouse IgG Fc. The concentration of crosslinking antibody corresponded to 50% of the original concentration of the total soluble protein from the previous step. The cells were incubated with crosslinking antibody for 15 min on ice and immediately transferred to 37 °C water bath for stimulation and downstream processing for phospho-flow cytometry.

### Phospho-flow cytometry

For T cell subset phospho-flow cytometry, primary human CD3+ T cells were initially stained with the following anti-human surface antibodies as per standard protocol: CD4-AF700 (clone RPA-T4), CD8-Pacific Blue (clone RPA-T8), CD45RA-VB605 (clone HI100), and CCR7-AF488 (clone G043H7). Next, the cells were washed and subjected to crosslinking stimulation. Bead-stimulated Jurkat cells or primary T cells stimulated by crosslinking were resuspended in fixation buffer (BioLegend) immediately after the end of each stimulation at 37 °C water bath. The cells were resuspended by vortexing and incubated with the fixative for 15 min at 37 °C water bath. Next, the cells were washed once with cold FACS buffer containing PBS and 2% FCS at 450 × *g* for 5 min and RT. The crosslinking antibody binding was quenched by resuspending the cells in 100 μl of 15-μg mouse IgG isotype (Thermo Fisher Scientific) prepared in PBS and incubation at RT for 15 min. Following another wash with FACS buffer, the cells were resuspended in 100% ice-cold methanol and stored at −20 °C overnight. Next, the cells were washed twice with cold FACS buffer at 1000 × *g* for 5 min and RT and stained with anti-human CD3ζ pTyr-142 antibody (clone K25-407.69 from BD Bioscience) for 45 min at RT in the dark. After two more washes in FACS buffer at 1000 × *g* for 5 min and RT, the cells were acquired on BD LSRII flow cytometer. Single stained UltraComp beads (Thermo Fisher Scientific) were used to adjust the voltages and for compensation. The data were analyzed in FlowJo (FlowJo, LLC) and fluorescence minus one controls for each fluorescent channel were used to set up the analysis gates.

### Western blotting

Following 37 °C water bath stimulation, Jurkat T cells were placed on ice, resuspended with 900 μl of ice-cold PBS, and centrifuged for 5 min at 400 × *g* and 4 °C. The cell pellets were resuspended in cold RIPA lysis buffer, containing 1 mm sodium orthovanadate and complete Mini, EDTA-free protease inhibitors (Roche). The cells were placed on a rotator and lysis was carried out at 4 °C for 30 min. The lysates were centrifuged for 10 min at 12,000 × *g* and 4 °C, and 30 μg of each lysate were resuspended in reducing Laemmli buffer, boiled at 95 °C for 10 min, and run on SDS-PAGE. Following protein transfer for 30 min at 25 V, the nitrocellulose membrane was blocked with 5% BSA in PBS containing 0.05% Tween-20 and blotted overnight with 2 μg/ml total phosphotyrosine antibody (clone 4G10, EMD Millipore) prepared in PBS containing 0.05% Tween-20 containing 2% BSA. The membrane was developed with 0.1 μg/ml IRDye secondary fluorescent antibody and acquired on Odyssey CLx Imaging system. To determine PAK phosphorylation, 5 × 106 Jurkat T cells from each cell line were washed once with cold PBS and resuspended in cold RPMI. The cells were stimulated for 5 min with Dynabeads at 37 °C in a water bath as indicated above. Next, the samples were treated with Calyculin A (Cell Signaling Technology 9902S) at 100 nm for 25 min at 37 °C, washed once with cold PBS, centrifuged at 500 × *g*, and lysed with RIPA buffer supplemented with PhosStop (Roche 04906837001) for 1 h at 4 °C. Following 10-s sonication and centrifugation at 12,000 × *g* for 10 min at 4 °C, the samples were blotted using Phospho-PAK1 (Ser-199/204)/PAK2 (Ser-192/197) Antibody (Cell Signaling Technology 2605). Blots were imaged and analyzed using the Li-COR Odyssey software.

### Lentiviral transduction and Jurkat cell cloning

For lentiviral production, full-length PD-1 was cloned into pCDH-CMV-MCS-EF1α-GreenPuro vector (System Biosciences) and cotransfected with pMD2G envelope and psPAX2 packaging plasmids in HEK293T cells using SuperFect transfection reagent (Qiagen). Two million Jurkat cells were lentivirus transduced by spinoculation at 800 × *g* for 30 min at 32 °C. For the generation of PD-1 expressing Jurkat clone, PD-1–transduced Jurkat cells were stained with anti-human PD-1 (clone EH12.2H7, BioLegend). Single cells were sorted on BD FACSAria in 96-well plates containing 100 μl of filtered media from Jurkat cell cultures and 100 μl of complete RPMI.

### Confocal microscopy

To evaluate conjugate formation by confocal microscopy, PD-1–transduced or untransduced Jurkat cells were transfected with DMRIE-C reagent and mCherry-LifeAct-7 plasmid (Addgene) according to manufacturer's recommendations. On next day, 1–2 × 10^6^ Raji B cells were coated with 2 μg/ml SEE in FCS-free RPMI for 2 h at 37 °C, 5% (v/v) CO_2_. Next, 2 × 105 Raji B cells prepared in 200 μl were mixed with 100 μl of untransduced Jurkat T cells or PD-1–transduced Jurkat clone. The coculture was placed on a glass bottom culture dish (MatTek Corporation) and rested at 37 °C for 30 min to allow conjugate formation. Following, confocal images from each stimulation were acquired on Zeiss 710 confocal microscope and analyzed with ZEN software.

### Rap1 activation assay and Western blotting

Activated Rap1 was detected by using the GSH S-transferase pulldown assay, as previously described (12). Cell lysates were separated by using Tris-glycine PAGE and transferred to nitrocellulose filters. Blots were blocked, incubated with the primary antibodies at 4 °C, and then washed and incubated for 45 min at room temperature with conjugated secondary antibodies (Li-COR Biosciences). Immunoreactive bands were visualized using the Odyssey Imaging System (Li-COR Biosciences).

### RT-qPCR analysis of IL-2 and IFN-γ gene expression

Total RNA was extracted using the RNeasy Plus Mini Kit (Qiagen). RNA (500 ng) was used for cDNA synthesis using SuperScript II First Strand Synthesis (Invitrogen). Human IL-2, IFN-γ, and HPRT Taqman primer/probes were used for all Taqman gene expression assays with the Taqman Universal PCR Master Mix (Applied Biosystems). Quantitative gene expression analyses were performed with Applied Biosystems 7300 Real-Time PCR. Gene expression was analyzed by the ΔΔCt method.

### Statistical analysis

GraphPad Prism software and ggpubr library in R were used for statistical analysis. Unpaired Student's *t* test was used to compare differences between the means of two groups, and a two-tailed *p*-value ≤0.05 was considered statistically significant, where *p < 0.05, **p < 0.01, and ***p < 0.001. To compare the effects of different treatments on tumor volume, we used repeated measures two-way analysis of variance and Tukey's multiple comparisons test with individual variances computed for each comparison.

## Data availability

The raw phosphoproteomic data are available at MassIVE, RRID:SCR_013665. The MS raw files are accessible under MassIVE ID: MSV000084813.

10.13039/100000060HHS | NIH | National Institute of Allergy and Infectious Diseases (NIAID) (AI125640-01) to Adam Mor
10.13039/100008637National Cancer Institute (NCI) (CA231277-01A1) to Anna S. Tocheva, and Adam Mor
10.13039/100008887Eppley Foundation for Research (Eppley Foundation) (-) to Anna S. Tocheva
