# Supplementary material for: Quantitative phosphoproteomic analysis reveals involvement of PD-1 in multiple T cell functions
Source: J Biol Chem. 2021 Jan 13;295(52):18036–50. doi: 10.1074/jbc.RA120.014745 (PMC7939457; doi:10.1074/jbc.RA120.014745)
Supplement: Supplementary file 1 [file mmc1.zip › 161394_1_supp_613103_qf95yf.pdf]

SUPPORTING INFORMATION

Quantitative phosphoproteomic analysis reveals involvement of PD-1 in multiple T cell functions

Anna S. Tocheva, Michael Peled, Marianne Strazza, Kieran R. Adam, Shalom Lerrer, Shruti Nayak, Inbar Azoulay-Alfaguter, Connor J.R. Foster, Elliot A. Philips, Benjamin G. Neel, Beatrix Ueberheide, Adam Mor

Figure legends to supporting excel tables.

Table S1. Log_2-transformed normalized ion intensities and linear regression analyses of phosphosites identified following 30 second stimulation of Jurkat cells. Of note, column G displays assigned study ID number, while the position of the phosphorylated residues is shown in column B.

Table S2. Log_2-transformed normalized ion intensities and linear regression analyses of phosphosites identified following 5-minute stimulation of Jurkat cells. Of note, column G displays assigned study ID number, while the position of the phosphorylated residues is shown in column B.

Table S3. Differentially phosphorylated S/T/Y sites from both time points curated by comparison. Of note, columns E, N and W display assigned study ID number and not the position of the phosphorylated residues.

Table S4. Functional annotation of differentially phosphorylated sites in response to PD-1 ligation.

Table S5. Kinase-substrate predictions.

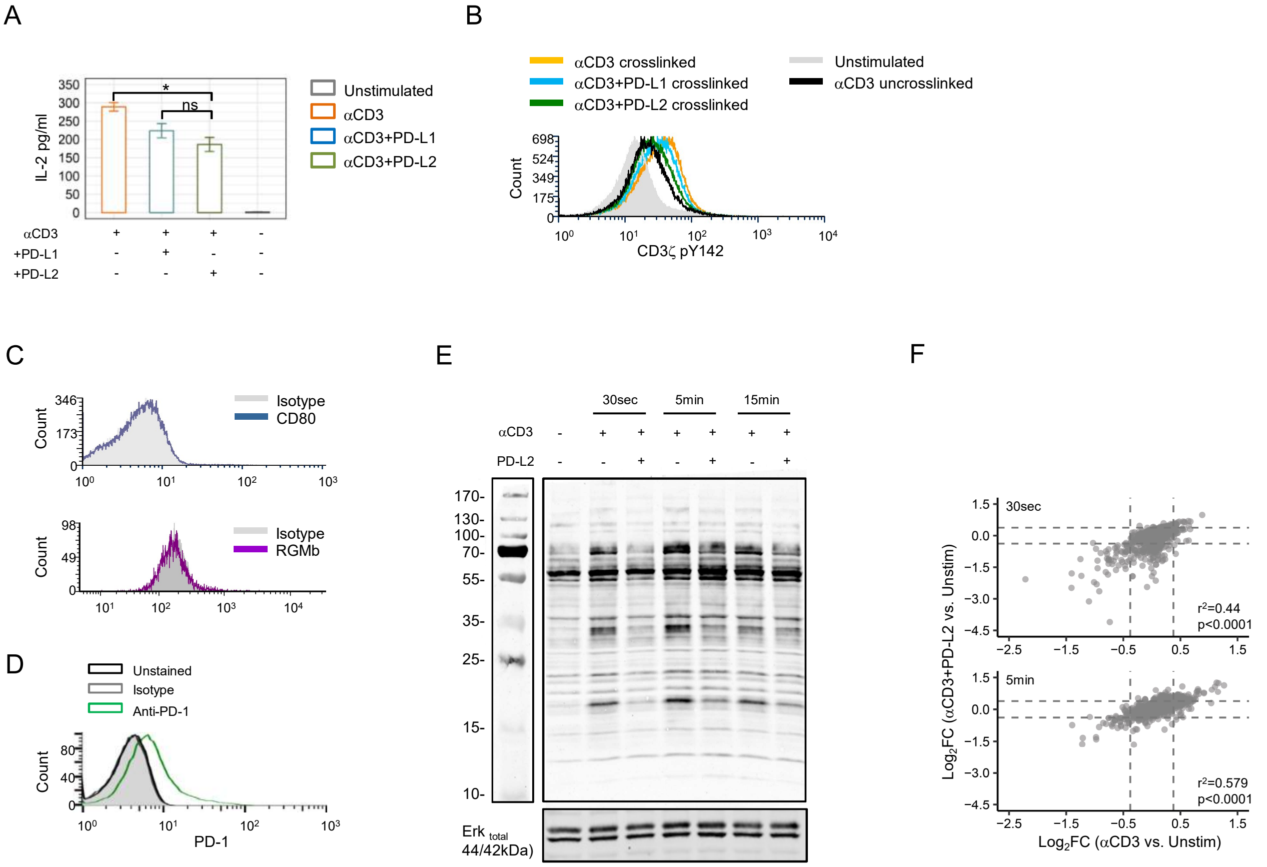

Figure S1. Experimental system validation. (A) Overnight IL-2 secretion from Jurkat cells stimulated with αCD3, αCD3+PD-L1 or αCD3+PD-L2 beads. Three independent experiments were performed and the data show mean±SD of one representative experiment performed in triplicate. (B) Jurkat T cell pre-treated with 0.5ug/ml PHA overnight in order to induce PD-1 expression were subjected to 5 minute crosslinking stimulation as indicated. Following, CD3ζ Y142 phosphorylation level was determined by flow cytometry. (C) Jurkat T cell surface expression of CD80 and RGMb. (D) PD-1 surface expression in Jurkat cells. (E) Total phosphotyrosine blot of Jurkat cell lysates following stimulation with beads coated with αCD3 or αCD3+PD-L2 for the indicated time points. (F) Scatter plots and linear regression analysis of phosphosite Log_2FC.

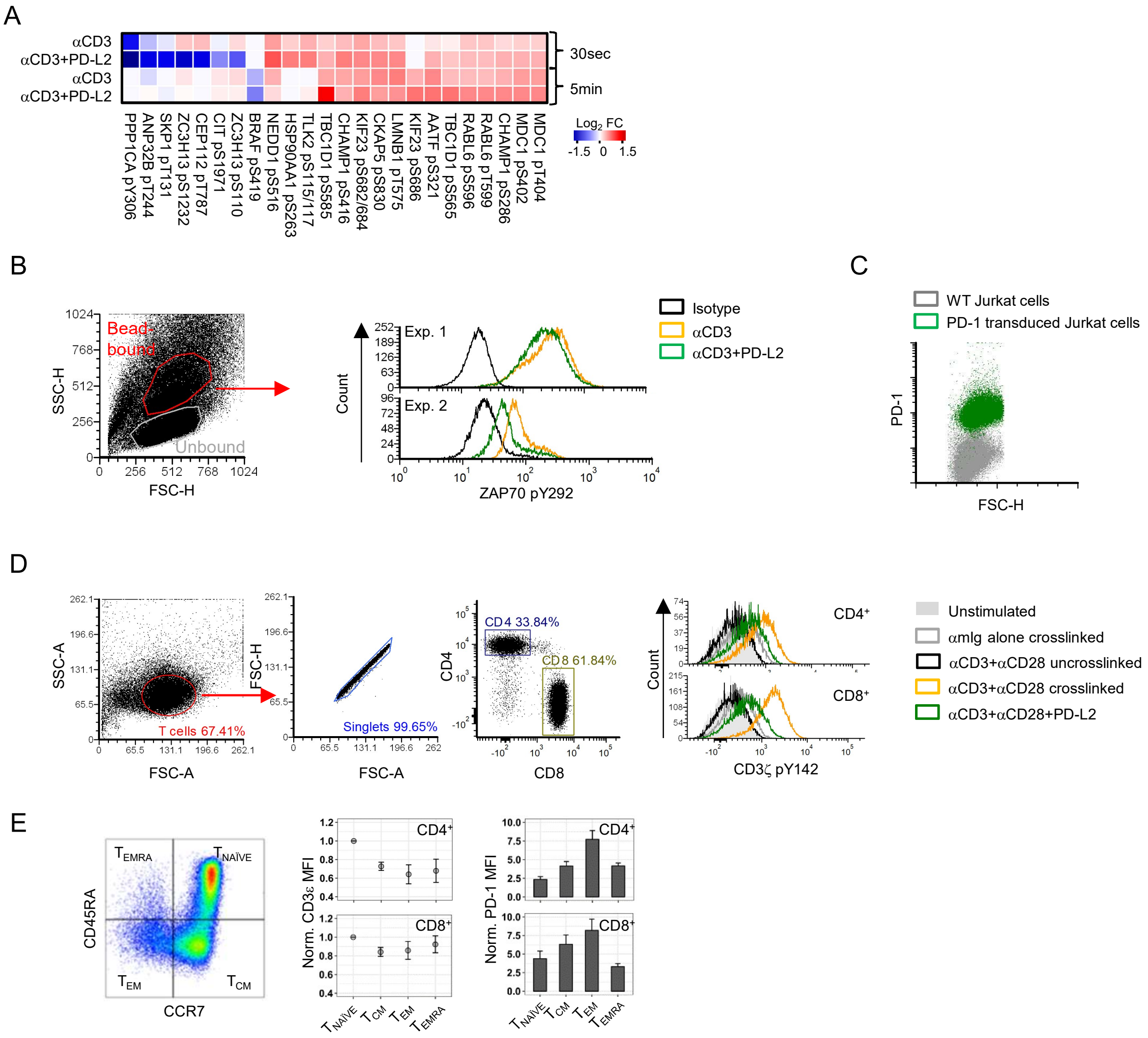

Figure S2. PD-1 targets cell cycle and proximal TCR signaling-related proteins leading to diminished cytokine production. (A) Heatmap of phosphosites within cell cycle-related proteins targeted by PD-1 showing Log$_2$FC values relative to the unstimulated control. (B) ZAP70 Y292 phosphorylation level from two independent experiments in wild-type Jurkat cells following 5 minute stimulation with αCD3 or αCD3+PD-L2 beads in the presence of soluble anti-human CD28. (C) PD-1 surface expression in Jurkat clone transduced with PD-1. (D) Gating strategy and representative histogram of CD3ζ pY142 dephosphorylation in primary human T cells following 5 minute stimulation by ligand crosslinking. (E) T cell subset gating strategy, CD3ε surface expression in T cell subsets normalized to CD3ε expression in naïve T cells and PD-1 MFI normalized to a fluorescence minus one control.

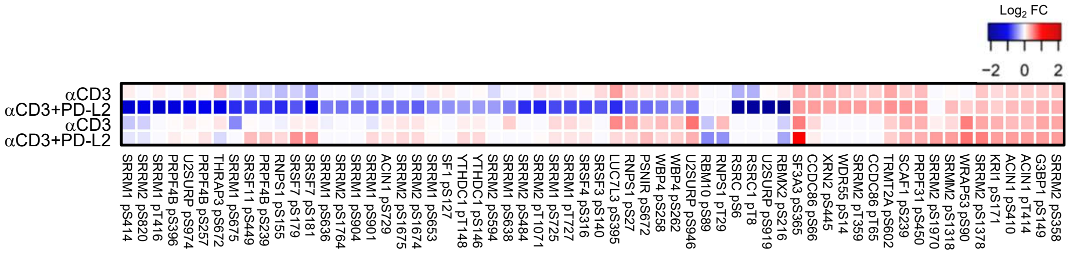

Figure S3. PD-1 ligation leads to significant S/T dephosphorylation within proteins mediating pre-mRNA processing and splicing. Heatmap of Log_2FC of normalized phosphosite intensities relative to the unstimulated control.
